# A tale of two comorbidities: Understanding the neurobiology of depression and pain

**DOI:** 10.4103/0019-5545.64586

**Published:** 2010

**Authors:** Meera Narasimhan, Nioaka Campbell

**Affiliations:** Research and Scientific Initiatives, Department of Neuropsychiatry and Behavioral Science, University of South Carolina School of Medicine; 1 General Psychiatry Training Program, Department of Neuropsychiatry and Behavioral Science, University of South Carolina School of Medicine

**Keywords:** Comorbidities, neurobiology, depression and pain

## Abstract

The comorbidity of chronic pain and depression has been consistently associated with a poor prognosis and greater disability in patients as compared to those suffering from each illness alone. This further has implications on significant financial costs to the patients and to our society. The biological underpinnings of major depression and chronic pain have considerable overlap in the areas of genetic, structural, functional, neuroendocrine and neurotransmitter functionality. Although the field has evolved in the past decade, more efforts should now focus on understanding the biological underpinnings of this shared comorbidity, while shedding light on treatment implications for these two devastating conditions.

## INTRODUCTION

There is a burgeoning interest in the mind-body connection in mental illness over the recent years. This is further substantiated by a growing body of evidence on the neurobiology of pain and depression given the cross talk between pain and affective pathways, outlining the interactions between affective, executive, cognitive processes and pain experiences. Depressed patients have a four times greater risk, than non-depressed patients, of a chronic painful physical condition (CPPC) while 69% of patients in primary care clinics present with somatic symptoms leaving depression unrecognized and undertreated.[1,2]

Current diagnostic criteria for major depressive disorder (MDD), established in the American Psychiatric Association’s Diagnostic and Statistical Manual of Mental Disorders, 4^th^ Edition, Text Revision (*DSM-IV-TR*), enumerates the presence of symptoms that occur across the physical, psychological and cognitive domains.[3] Depression encompasses physical causes and consequences (like appetite and sleep disturbance, fatigue, and chronic pain). Yet pain is not listed as a symptom of any mood disorder, and depressive and anxiety complaints are frequently marginalized in the list of symptoms required to meet criteria for a chronic pain disorder. In clinical practice, somatic complaints most commonly associated with depression include back pain, muscle, joint and limb pains, sleep disturbances, chest pain, dizziness and fatigue. In a study of primary care patients by Kroenke *et al*. the most common symptoms in patients with a mood disorder were back pain, musculoskeletal complaints like muscle, joint or limb aches and pains, sleep disturbances (insomnia), chest pain, dizziness and fatigue.[4] The likelihood of a mood disorder increased dramatically with increasing numbers of physical symptoms. The prevalence of a mood disorder increased from 2% for patients with zero to one symptom, to 60% for patients with nine or more physical symptoms.[4] A similar pattern was observed among patients with an anxiety disorder. Multiple or unexplained somatic symptoms may signify presence of a mood or anxiety disorder.

Chronic painful physical conditions (CPPC) are common in subjects with MDD and a recent international study has shown that the presence of a CPPC increased the frequency and severity of seven depressive symptoms including depressed mood, lack of interest, psychomotor agitation/retardation, weight gain, insomnia, fatigue, and impaired concentration.[5] Some physical symptoms of depression may be attributable to chronic pain rather than depressive illness, but, conversely, physical symptoms may be due to MDD rather than chronic pain. Clinical vigilance and discernment is necessary to determine whether physical symptoms indicate depression.

There is a bidirectional complex relationship between pain and depression with chronic pain as a predictor of subsequent major depression, and vice versa.[6] Co morbid pain and depression significantly complicate the treatment of each condition. The presence of physical symptoms in depression may affect response and remission to treatment and may pose a significant risk factor for relapse.[7,8]

This article is an attempt to review current scientific understanding of overlapping pathophysiological processes and circuitry of pain and depression that would help shed some light on the neurobiology and treatment implications that could potentially have significant relevance on clinical outcomes for our patients.

## MAGNITUDE OF THE PROBLEM

The co morbidity of chronic pain and depression has been consistently associated with a poorer prognosis and greater disability in patients as compared to those suffering from each illness alone.[9,10] The financial cost of these disorders is estimated in the billions, while the psychological and social effects are more difficult to quantify.[9] The lifetime prevalence of clinical depression in the United States is approximately 17%, while the rate of chronic pain ranges from 9-33%.[9–11] When in combination, prevalence rates of depression in patients with persistent pain have been reported to range from 30-60%.[11–13] At the very least, nearly one out of every three people in the community with persistent pain will report depression.[11] Patients with more severe and persistent pain have also been shown to have a higher risk for more severe depression and a greater association with suicidality.[14] Chronic pain may also be a predictor of subsequent development of major depression, while depression may be an indicator for the development of chronic pain.[15] Research has shown that psychotropic agents used to treat major depression such as the serotonin norepinephrine reuptake inhibitors (SNRIs) are also effective in reducing the symptoms of various pain conditions.[9,10,12] This evidence encourages the growing consideration of the neurobiological link between these disorder spectrums. Understanding the pathophysiological processes of pain, depression and the overlap of these illnesses may provide direction for research, assessment and future treatment modalities.

## BIOLOGY

The biological mechanisms underlying the link between pain and depression are heterogeneous.[15–17] Areas of interest include genetic, cellular, structural, functional, neurotransmitter and neuroendocrine alterations that exist in both disorders.

The complexity of genetic determinants for chronic pain and depression involves the multiple polymorphic nature and varying contribution of the genes which may contribute to these disorders. Although many genes have been implicated in major depression, the genes regulating serotonergic function such as the serotonin-transporter-linked promoter region 5HTTPR or the serotonin 2A (5-HT2A) are consistently identified.[16,18] Other genes involved in regulating neurotrophic factors which affect cellular resilience and neuroplasticity may have an additive role in the determination of developing these illnesses.[19] Catechol-O-methyl transferase (COMT) and monoamine oxidase (MAO) gene polymorphisms perhaps affect the response to other forms of psychological stress by altering the reaction of the hypothalamic-pituitary axis.[20] Genes which have also been shown as indicators for pain states include the 5HTTPR, 5HT2A and COMT polymorphisms.[21,22] These shared genetic polymorphisms may represent areas of vulnerability for both depression and pain syndromes.[15]

At the cellular level, inflammatory signals from distress or pain are conveyed primarily from the periphery to microglia.[23,24] In major depression, an aberrant neuronal-glial relationship may contribute to cellular toxicity from the diminished capacity of protective neurotrophic factors, the compromise of oxidative regulation, and the resultant accumulation of proinflammatory cytokines and glutamate.[25] Depression and chronic pain have both been associated with dysfunctions in the regulation of glutamate.[15] Studies now suggest a similar dysfunction of the neuronal-glial relationship and inflammatory signaling may exist in chronic pain states such as neuropathic pain and fibromyalgia.[26]

Neuroimaging studies have allowed for examination of the areas of the brain which are structurally or functionally aberrant in both pain and depressive syndromes. The dorsolateral prefrontal cortex (DLPFC) is the area of the brain which regulates influence over limbic structures and is a crucial part of the executive function network.[27] Studies have shown that patients with major depression may have reduced activity and decreased gray matter volume in the DLPFC.[28] This same structural change of reduced gray matter volume in the DLPFC has been correlated with neurocognitive deficits in patients with fibromyalgia.[29] Functional magnetic resonance imaging (MRI) studies suggest that the same CNS pathways may exist for both depression and physical pain symptamatology as demonstrated by the mediation of negative affect via the dorsal anterior cingulate in response to both physical and emotional distress.[15] Other functional and structural changes that overlap among depression and pain syndromes such as fibromyalgia include excessive activation of the amygdala and disruption/volume loss in the hippocampal regions.[30,31] Functional regulation of the nucleus accumbens and ventral tegmental area is disrupted in both chronic pain states and major depression.[32] These changes in the limbic system may lead to further autonomic dysfunction and sympathetic responses that combine with proinflammatory reactivity which can worsen mood or pain symptoms.[33]

Neuroendocrine abnormalities such as dysfunction in the hypothalamic-pituitary-adrenal (HPA) axis have been recognized in both depression and chronic pain disorders, such as the inability to suppress cortisol as seen in patients with fibromyalgia.[34] Physical or psychological stress can increase serum glucocorticoid concentrations which can produce atrophic changes in hippocampal sub regions and volume reduction that is sometimes seen in depression. In addition, atypical depression may be characterized by decreased serum cortisol which is often seen in other disorders such as fibromyalgia or chronic fatigue syndrome.[12] The signal strength of the hormonal response depends on the effectiveness of the signaling and not on hormonal levels alone.[15] Research has shown that the glucocorticoid receptors are less active in depressed patients, causing a failure of signaling even in the presence of increased cortisol levels.[35] Regardless of the debate between excessive or impaired glucocorticoid signaling, the loss of normal diurnal cortisol patterns or HPA axis has been a consistent finding in the symptoms of pain and depression.[34,36] Disturbances in autonomic nervous system activity have been shown in depressive and pain syndromes, comprised of an increased sympathetic and diminished parasympathetic tone.[15] These common abnormalities of the HPA and autonomic systems for both pain and depression tend to activate the same inflammatory pathways, perpetuating dysfunction in neuroimmune processes.[37]

The role of neurotransmitters, peptides and endogenous opioids continues to be a focus of research in chronic pain syndromes and depression. Serotonin, a neurotransmitter well known for its usefulness in antidepressants, has been shown to have antinociceptive and pronociceptive effects depending on the receptor subtype and region of the central nervous system.[11,15] Descending inhibitory pathways within the CNS reduce activity of nociceptive pain neurons by release of serotonin and norepinephrine (NE). However, serotonin is also a player in descending facilitation pathways, which suggest how an SSRI alone is not always effective in the treatment of major depression.[11] NE, which is decreased in the cerebrospinal fluid (CSF) in some depressed patients, participates alongside serotonin in the sensitizing of peripheral C-fiber axons in neuropathic pain.[11,38] In addition, if descending NE inhibition is deficient, it may not mask irrelevant nociceptive input, leading to perceived pain that is otherwise ignored.[11] Substance P is an active neuropeptide in the CNS and gastrointestinal system that leads to pain, motility, anxiety, and inflammation as part of the stress response.[11,39] NE may inhibit substance P, thus low NE could indirectly cause more nociception.[11] Altered endogenous opioid signaling or decreased receptor availability may represent a role in the modulation of pain and depressive symptoms.[40] Finally, dysfunctions in glutamate and GABA transmission have been demonstrated in the pathogenesis of depression and pain syndromes.[15] Glutamate is the primary excitatory neurotransmitter for linking limbic and cortical areas as well as inhibiting or facilitating pain transmission in the CNS.[15] GABA, which plays a role in pain transmission and mediation of neuropathic pain, is associated with major depression in the context of reduced plasma GABA levels.[41,42]

The biological underpinnings of major depression and chronic pain have considerable overlap in the areas of genetic, structural, functional, neuroendocrine and neurotransmitter functionality. The pathology that is similar between these illnesses therefore results in similar phenomenology that is seen in clinical practice.

## CLINICAL SIGNIFICANCE AND CONCLUSIONS

There is sufficient evidence to suggest that nearly 30-60% of patients with depression also suffer from pain syndromes.[13] More often than not those with chronic pain also meet criteria for a mood disorder diagnosis. Studies have distinctly elucidated a significant overlap in the pathophysiological process of pain and depression.

Stress seems to play a role as a precipitant and a perpetuator in both pain and depression. In addition, both of these syndromes seem to share neurobiological underpinnings including the rubric of genes, brain circuitry, dysregulation of neurochemistry (i.e. monoamines, peptides, opioids, GABA, glutamate), inflammatory processes, neuroendocrine and neurotrophic signaling. Neuroanatomic regions that play a role in both these syndromes include the limbic and paralimbic prefrontal cortical areas – amygdala, hippocampus, insula, anterior cingulate cortex (ACC), ventromedial prefrontal cortex (vmPFC) as well as more "cognitive" and integrative brain areas.

There is a bidirectional relationship between pain and depression with the symptoms of one worsening the other. These co morbid conditions also complicate treatment response and increase risk for relapse, thereby warranting the need to address both of these problems concurrently.[8,43,44] Consistent with this, timely treatment of MDD may optimize the chance of remission and minimize structural cognitive changes.[45–49] Full and sustained remission may prevent future recurrences.[50] Co morbidity of MDD and pain may pose a significant barrier to early and appropriate diagnosis of MDD and delay treatment and subsequent benefits of remission.[1,51] This overlapping pathology results in similar phenomenological manifestations. Although the field has evolved in the past decade, more efforts should now focus on understanding the biological underpinnings of this shared co morbidity, while shedding light on treatment implications for these two devastating conditions.
